# A History of Surgery, from Its Origin to the Present Time

**Published:** 1782

**Authors:** 


V. *Hiſtoire de la Chirurgie, depuis ſon origine juſqu'à nos jours*, i. e. *A Hiſtory of Surgery, from its origin to the preſent time. By* M. Dujardin, *Member of the Royal Academy of Surgery, and of the Imperial Academy of Naturæ Curioſi.* Vol. I.

4to.

4to. Paris. 577 pages, with 4 copper-plates.

THIS learned and elegant volume, which is all the author lived to finiſh of his intended work, brings down the hiſtory of ſurgery to the time of Celſus. It contains four books. In the firſt the author treats of the ſtate of ſurgery among the moſt ancient nations, ſuch as the Jews, the Phœnicians, the Aſſyrians, the Ægyptians, and the Greeks. The delivery of the fœtus, as having been one of the earlieſt operations of ſurgery, firſt fixes his attention; after which he proceeds to the method of embalming dead bodies, as practiſed by the Ægyptians. The book cloſes with an account of the ſurgery of the Chineſe and Japaneſe, amongſt whom this art is ſtill in a ſtate of rudeneſs and ſuperſtition.

The ſecond book contains the ſurgery of the Greeks, and ſome other nations, to the time of Hippocrates. The ſtate of the art under that venerable father of phyſic, and his ſucceſſors, to the time when it began to be introduced amongſt the Romans, is the ſubject of the third book; and the fourth affords a view of the progreſs of ſurgery among the latter before and after after the arrival of the Greek and Arabian ſurgeons, and concludes with a very full account of the ſurgery of Celſus.

Prefixed to the whole is an introduction, in which the learned author gives an account of the ſuperſtitious practices that prevailed in ſurgery in the earlier ages, together with a view of the origin and hiſtory of ſome particular cuſtoms, ſuch as circumciſion and caſtration, which it was neceſſary to mention in a work of this nature.

To this conciſe view of the general plan of the volume, we ſhall add a few detached paſſages from different parts of it, that our readers may be enabled to form an idea of the author's manner.

*Origin of the actual Cautery.*—"The Greeks "adopted this practice very early, and ſeem "to have borrowed it from the Egyptians "or the Ethiopians. Theſe laſt were ac-"cuſtomed to burn the forehead of their "children the day they were born (Bartholin, "*de puerperis veter.*) and the Etruſcans did the "ſame to the occiput. This remedy was in "great uſe among the Aſclepiades, as appears "from a paſſage in Jamblicus (*de vita Pythagoræ*,

"cap.

" cap. 29,) and from the writings of Hippo-
" crates. Befides, from time immemorial, it was
" in ufe among different nations. The Lybians
" (*Herodotus*, *lib.* iv.) a people of Africa, efpe-
" cially thofe who led a paftoral life, held it in
" high eftimation. When their children had
" attained their fourth year, they conftantly
" cauterized them, either in the veins at the top
" of the head, or in thofe of the temples, with
" greafy wool, in order to preferve them from
" the phlegm and pituita which they fuppofed
" to flow from the brain. When this operation,
" as fometimes happened, excited a fort of fpafm,
" they calmed it by fprinkling the patient with
" goat's urine. Thefe people, who, in general,
" were a robuft race, attributed the firmnefs of
" their health folely to this precaution.

" The Scythians, (Hippoc. *de aere*, &c.) a
" nation who difputed the antiquity of their
" origin with the Egyptians, were in the habit
" of applying the cautery to their fhoulders,
" arms, joints, breaft, and loins; and the reafon
" of this cuftom was the exceffive humidity and
" weaknefs of their articulations. It may be
" prefumed likewife, from an anecdote related
" by Pliny (*Hift. Nat. lib.* 26, c. 1.) that the
" ufe of the fame remedy among the Egyptians

" was

"was of a very ancient date. So early as the "time of the Emperor Claudius, when the "*Montagra* (a diſeaſe ſo called, becauſe it par- "ticularly attacked the chin) began to appear "at Rome, Egyptian phyſicians were ſent for "as being the moſt experienced in the treatment "of this complaint, which was common in their "country, and by theſe the application of fire "was recommended as the moſt ſucceſsful mode "of cure. This excellent remedy, which the "ſoftneſs of our manners has rendered ſo rare "among us, is ſtill in frequent uſe in Lapland "for pains of the joints, and likewiſe in other "countries where the art is yet in its infancy."

*Homer.*—"We ought here to pay ſome "homage to the talents and learning of Homer. "Altho' he was not a phyſician, yet his poems "may be ſaid to contain almoſt all the ancient "Phyſic of the Greeks. We find in them a "variety of information concerning the Anatomy, "and Surgery of thoſe remote times, which we "ſhould in vain ſearch for elſewhere. Without "giving into the extravagant encomiums of ſome "of his admirers, we cannot but acknowledge "that he poſſeſſed a degree of knowledge that "muſt aſtoniſh us, when we conſider the time "in which he lived. He is the Hiſtorian of the

" arts, and the Painter of ancient manners, and

" yet his poetry loſes nothing either of its ſtrength

" or its beauty. His deſcriptions ſhew that the

" Anatomy and the Surgery of his time were

" familiar to him. He knew that a wound of

" the vena cava was ſpeedily followed by death.

" We are indebted to him for a deſcription, that

" is ſufficiently ſatisfactory, of the tendon of

" Achilles, ſo called, probably, becauſe, among

" the ancients the name of *Achilles* was given to

" every thing that had any extraordinary ſtrength

" or virtue. In a word, we may obſerve all the

" exactneſs and preciſion that can be expected

" from thoſe early ages, in the account he gives

" of the method of treating wounds, ſuch as the

" manner of waſhing them, ſtopping the blood,

" extracting from them arrows and darts, and

" applying to them ſuitable remedies."

*Venæſection.*——" The ancients imagined that

" young and old people were equally incapable

" of ſupporting venæſection, and that if a preg-

" nant woman was let blood, ſhe was in danger

" of abortion, which might be true from the

" great quantity of blood they uſed to take away.

" Theſe profuſe evacuations, which, in certain

" caſes, ſeemed to have a very favourable effect,

" at length fell into diſrepute becauſe the practice

"was abused, and then they began to draw off

"in two days the quantity they had before been

"accustomed to take away in one. In particular

"circumstances the same good effects were not

"obtained, but then they were not exposed to the

"same dangers. As these moderate evacuations

"occasioned no obvious ill effect, they insensibly

"ventured to bleed children, old persons, and

"pregnant women; and as the quantity of blood

"taken away was proportioned to the strength

"of the patient, this practice was justified by

"the event. When they had begun, contrary

"to the advice of Hippocrates (Aph. 31. sect. 5.)

"to bleed pregnant women, a prudent use of

"this salutary remedy enabled them to cure

"those acute diseases, which on his authority

"had been deemed mortal*. Thus by con-

"vincing themselves of the falsity of one apho-

"rism, they discovered that of another, which

"could be true only in proportion as they were

"strict observers of the first."

*Cupping.*——"Another method of drawing
"blood, and which was in frequent use among
"the ancients, was by cupping. In the time

* *Mulierem gravidam morbo quopiam acuto corripi, lethale.* Aphor. 30. sect. 5.

"of

" of Celſus there were two kinds of inſtruments

" for this purpoſe. One of theſe was of copper,

" and the other of horn. The latter was nothing

" more than the horn of an animal, with a hole

" bored through its extremity, in order that the

" air might be drawn out by ſuction. When

" the inſtrument was fixed, this opening was

" cloſed with a little wax. This was the moſt

" ſimple kind of cupping inſtrument, and of

" courſe the firſt that was in uſe. Thoſe of cop-

" per pretty much reſembled our cupping glaſſes,

" and were applied with lighted lint. Chance

" rather than any preciſe knowledge of the rare-

" faction of the air, had led to this mode of

" applying cupping inſtruments ſo early as the

" time of Celſus."

M. Peyrilhe, a very ingenious Profeſſor of Surgery at Paris, who has undertaken to continue and complete this work, has lately publiſhed a ſecond volume, which is extremely well written, and of which we mean ſoon to give an account.

